# Human Immunodeficiency Virus (HIV) Treatment With Antiretroviral Therapy Mitigates the High Risk of Mental Health Disorders Associated With HIV Infection in the US Population

**DOI:** 10.1093/ofid/ofad555

**Published:** 2023-11-07

**Authors:** Djibril M Ba, Kathryn A Risher, Paddy Ssentongo, Yue Zhang, Qi Dai, Guodong Liu, Mamoudou Maiga, Xuehong Zhang, Brehima Diakite, Souleymane dit Papa Coulibaly, Lifang Hou, Douglas L Leslie, Vernon M Chinchilli

**Affiliations:** Department of Public Health Sciences, Penn State College of Medicine, Hershey, Pennsylvania, USA; Department of Public Health Sciences, Penn State College of Medicine, Hershey, Pennsylvania, USA; Department of Public Health Sciences, Penn State College of Medicine, Hershey, Pennsylvania, USA; Department of Medicine, Penn State Milton S. Hershey Medical Center, Hershey, Pennsylvania, USA; Department of Public Health Sciences, Penn State College of Medicine, Hershey, Pennsylvania, USA; Department of Medicine, Vanderbilt University, Nashville, Tennessee, USA; Department of Public Health Sciences, Penn State College of Medicine, Hershey, Pennsylvania, USA; Preventive Medicine Department, Northwestern University, Chicago, Illinois, USA; Department of Nutrition, Harvard T. H. Chan School of Public Health, Boston, Massachusetts, USA; Department of Medicine, Brigham and Women's Hospital, Harvard Medical School, Boston, Massachusetts, USA; Faculty of Medicine and Odontostomatology, University of Sciences, Techniques, and Technologies of Bamako, Bamako, Mali; Faculty of Medicine and Odontostomatology, University of Sciences, Techniques, and Technologies of Bamako, Bamako, Mali; Preventive Medicine Department, Northwestern University, Chicago, Illinois, USA; Department of Public Health Sciences, Penn State College of Medicine, Hershey, Pennsylvania, USA; Department of Public Health Sciences, Penn State College of Medicine, Hershey, Pennsylvania, USA

**Keywords:** ART, HIV, mental health, real-world data, US

## Abstract

**Background:**

Whether treatment of human immunodeficiency virus (HIV) with antiretroviral therapy (ART) is associated with lower risk of mental health disorders (MHDs) among people with HIV (PWH) remains unknown. We aim to determine the association between HIV and MHDs and whether ART alters the risk of MHDs among PWH in the US adult population.

**Methods:**

We conducted a real-world study using the Merative MarketScan claims database (2016–2020), identifying individuals with HIV (diagnosed using *International Classification of Diseases, Tenth Revision, Clinical Modification* codes) and those without HIV. A multivariable stratified Cox proportional hazard regression model was conducted to examine the association of HIV treatment status with MHDs, adjusting for potential confounders. Additionally, we sought to determine the effect modification of ART on the relationship between living with HIV and MHDs.

**Results:**

A total of 313 539 individuals, with a mean age of 44.2 (standard deviation, 11.4) years, predominantly males (81.2%), residing in the South region of the US (50.9%) were included in the present analysis. During 671 880 person-years of follow-up, 46 235 incident MHD cases occurred. In the multivariable Cox proportional hazard model, living with HIV was associated with higher risk of incident MHDs. Relative to those without HIV, the adjusted hazard ratio was 1.85 (95% confidence interval [CI], 1.79–1.92; *P* < .001) for those with HIV on treatment, and 2.70 (95% CI, 2.59–2.82; *P* < .001) for those with HIV without any treatment. Stronger associations between HIV and MHDs were observed in men relative to women, among those aged 18–34 years relative to those aged 55–63 years, and among those with no overweight/obesity relative to obese individuals (*P*_interaction_ < .001 for all).

**Conclusions:**

HIV was associated with an increased risk of developing MHDs. However, HIV treatment mitigated the risk.

Human immunodeficiency virus (HIV) remains a public health problem in the United States (US). According to the Centers for Disease Control and Prevention (CDC), there were an estimated 1.2 million people with HIV (PWH) in the US at the end of 2019 [1]. While much progress has been made, HIV remains incurable; however, antiretroviral therapy (ART) is effective in controlling disease progression, improving quality of life and achieving near-normal life expectancy [2, 3]. ART is a treatment regimen typically consisting of a combination of 3 or more antiretroviral drugs [3]. The US has made significant strides in HIV prevention, treatment, and care since the beginning of the epidemic 40 years ago [4] with a plan for ending the HIV epidemic, which is a federal effort designed to reduce new HIV infections in the US by 90% by 2030 [5].

Mental health disorders (MHDs), including major depression, are a significant public health problem and serious medical illness in the US [6, 7]. They are key contributing factors to the global burden of disease and are associated with increased rates of chronic diseases, suicide attempts, medical costs, disability, and impaired function [8–10]. According to the National Institute of Mental Health, nearly 1 in 5 US adults lives with mental illness, representing an estimated 52 million individuals in 2019 [11]. Every year, about 19 million American adults (9.5% of the adult population) suffer from a depressive illness [12]. According to a previous review, mental health illnesses are one of the most common comorbidities among PWH [13].

There have been few reported epidemiological studies on the association between HIV and MHDs [14]. However, these studies had several limitations such as not assessing the impact of HIV treatment on the risk of MHDs, or not including all categories of MHDs. Furthermore, these studies did not examine whether PWH who received ART treatment had modified risk of developing MHDs. For example, a study conducted by Mirza et al was limited to only US military personnel and had an almost exclusively male study population (97% of PWH were men). The study reported that about 56% of service members with HIV had higher risk of developing MHDs compared to those without HIV [15]. Another study conducted in the US found that in men who have sex with men (MSM) with HIV, sleep disturbance was associated with significant increases in depression compared to MSM without HIV [16]. A recent study conducted in the United Kingdom by Gooden et al suggested that PWH had higher risk of developing composite mental illness, depression, anxiety, and severe mental illness compared with persons without HIV [17]. To the best of our knowledge, no study has yet used a large insured US population to investigate whether ART mitigates the risk of MHDs among PWH. We aim to address this gap in the literature to examine the association between HIV and MHDs and whether ART is associated with an altered risk of developing MHDs among PWH in the US adult population.

## METHODS

### Data Source

This analysis was conducted using prospectively collected the Merative MarketScan Commercial Claims and Encounters database from 2016 to 2020. The Merative MarketScan Research Databases is one of the largest and longest nationwide longitudinal claims databases used for healthcare research that contains data on >275 million unique de-identified patients [18]. The MarketScan databases include covered employees and family members from large employers and health plans across the 50 states of the US and the District of Columbia [19]. Longitudinal tracking of detailed patient-level healthcare claims information provides comprehensive data, including demographic characteristics such as age, sex, geographic locations, inpatient and outpatient medical information, detailed prescription drug information, and financial information [19]. The MarketScan Research Databases fully comply with the Health Insurance Portability and Accountability Act of 1996 (HIPAA) [19]. The MarketScan database has been widely used in large epidemiologic outcomes research and health economic studies [20, 21]. The protocol of this study was reviewed and received a determination of non–human subjects research by the Penn State Institutional Review Board. The individual informed consent requirement was waived for this secondary analysis of de-identified data.

### Cohort Derivation and Assessment of Exposure

We identified PWH aged 18–63 years using *International Classification of Diseases, Tenth Revision, Clinical Modification* (*ICD-10-CM*) codes [22] (Supplementary Table 3) from 1 January 2017 through 31 December 2019. We had a very large number of persons without HIV in the database (>30 million); therefore, for each stratum of PWH by birth year and sex, we randomly selected 10 times as many persons without HIV as PWH. For these non-HIV patients, we randomly assigned a pseudo–index date from 1 January 2017, to 31 December 2019. Both PWH and persons without HIV did not have *ICD-10* diagnosis codes of MHDs prior to the index date. PWH and persons without HIV displayed a minimum of at least 12 months of continuous enrollment prior to the index date and at least 12 months of continuous enrollment after the index date. We identified a total of 37 329 PWH and 373 290 persons without HIV before excluding those with prevalent MHDs. After excluding individuals with prior diagnosis of MHDs, a total of 26 410 PWH and 287 129 without HIV diagnosis were included in the current analysis. We identified 17 830 PWH with any prescription of ART-related medications prior to the onset of MHDs (Supplementary Table 4) and 8580 patients without treatment. We used the earliest date of receiving HIV treatment as the index date for PWH with treatment and the earliest date when an HIV diagnosis code was observed for PWH without treatment in the current analysis.

Participants were divided into 3 categories in our primary analysis, according to HIV and treatment status: persons without HIV, PWH with treatment, and PWH without treatment.

### Assessment of Outcomes

The primary outcome was the composite of any MHDs, defined using *ICD-10* codes for mental health diagnosis or substance use disorders as done in previous studies [23, 24] (Supplementary Table 3) that occurred after index dates during the follow-up period. As the secondary outcomes (psychotic disorder, major depression, other mood disorder, anxiety, and related disorder, and other mental health conditions), the association of HIV with these individual MHD groups also was explored.

### Assessment of Potential Covariates

Demographic data on age (years), sex (male/female), place of residence (urban/rural), and US census region (South, West, Midwest, Northeast) were extracted directly from the MarketScan database. Based on a comprehensive literature review, the following potential confounding factors were identified at baseline (eg, during the 12 months prior to the index date), using their corresponding *ICD-10-CM* codes (Supplementary Table 3): overweight/obese, nonalcoholic fatty liver disease (NAFLD), ischemic heart disease, congestive heart failure (CHF), hypertension, diabetes, chronic kidney disease (CKD), dyslipidemia, and stroke, with each categorized as (yes/no).

### Statistical Analysis

We calculated the person-time of follow-up for each participant following the index date to the first occurrence of an outcome of interest (MHD), end of enrollment, or end of the study period (31 December 2020), whichever took place first. HIV status was deemed as the primary exposure. Descriptive statistics were calculated to summarize patient characteristics, stratified by HIV status, and were presented as age-adjusted percentages (for categorical variables) and means and standard deviations (for continuous variables), as applicable. An initial stratified Cox proportional hazard regression model adjusting for age and sex was applied to calculate the hazard ratio (HR) and 95% confidence interval (CI). We then ran a parsimonious multivariable stratified Cox regression model to adjust for all potential confounding factors.

We conducted subgroup analyses by calculating the unadjusted incidence rates and corresponding 95% CIs per 1000 person-years (PY) of follow-up for the 2 cohorts within each subgroup.

Considering that several comorbid diseases frequently are associated with HIV, we conducted several sensitivity analyses to test the robustness of our results and to address the possibility of residual confounding. First, we performed an analysis based on propensity score strata (propensity score stratification method). Propensity scores were estimated from a logistic regression model using the covariates in the full model, to balance baseline data between PWH and without HIV. Second, we further excluded those taking antipsychotics, antidepressants, and antianxiety drugs. Third, we reran the analyses for individuals without any apparent comorbidities after excluding those with overweight/obesity, NAFLD, ischemic heart disease, CHF, hypertension, diabetes, CKD, dyslipidemia, and stroke. Fourth, models including interactions with HIV, specifically age (years) and sex (the 2 most important determinants for risk of HIV and MHDs), and obesity in relation to MHD risk were assessed by the −2-log likelihood ratio controlling for the same covariates above. Subgroup analyses were further conducted when a significant interaction was observed. The proportional hazard assumption was checked by log-log survival curve. Data were analyzed in SAS software version 9.4 (SAS Institute, Cary, North Carolina) and R software version 3.6.2 (R Foundation for Statistical Computing, Vienna, Austria) using a 2-tailed α level of .05.

## RESULTS

In total, 313 539 individuals (mean age, 44.2 [standard deviation, 11.4] years), predominantly males (81.2%), residing in the South region of the US (50.9%) were included in the present analysis. During 671 880 PY of follow-up, we identified 46 235 incident MHD cases. PWH, particularly those without treatment, had higher likelihood of having chronic conditions, such as overweight/obesity, diabetes, stroke, ischemic heart disease, and CHF relative to persons without HIV (Table 1). Log-log survival curves for checking the proportional hazard assumption for 3 groups were almost completely parallel and proportional hazard assumption was satisfied (Supplementary Figure 1).

**Table 1.** Age-Standardized Characteristics of the Study Population at Baseline, by HIV Status

Characteristic	HIV Status		
	No HIV (n = 287 129)	HIV With Treatment (n = 17 830)	HIV Without Treatment (n = 8580)
Age of patient, y, mean (SD)^a^	44.0 (11.4)	46.9 (10.7)	43.8 (11.9)
Male	233 697 (81.4)	14 661 (82.2)	6338 (73.9)
Urban	244 477 (85.1)	15 036 (84.3)	7312 (85.2)
Region of United States			
Northeast	40 347 (14.1)	3139 (17.6)	2472 (28.8)
Midwest	73 278 (25.5)	1812 (10.2)	868 (10.1)
South	145 053 (50.5)	10 436 (58.5)	4448 (51.8)
West	28 451 (9.9)	2443 (13.7)	792 (9.2)
Comorbidities			
Overweight/obesity	21 021 (7.3)	1144 (6.4)	716 (8.3)
NAFLD	3041 (1.1)	278 (1.6)	134 (1.6)
Hypertension	58 622 (20.4)	3544 (19.9)	1718 (20.0)
Diabetes	25 677 (8.9)	1625 (9.1)	839 (9.8)
Dyslipidemia	62 117 (21.6)	4288 (24.1)	1998 (23.3)
Stroke	1793 (0.6)	143 (0.8)	85 (1.0)
Ischemic heart disease	6542 (2.3)	414 (2.3)	210 (2.4)
Congestive heart failure	2025 (0.7)	162 (0.9)	98 (1.1)
CKD	2469 (0.9)	572 (3.2)	189 (2.2)

Data are presented as No. (%) unless otherwise indicated and are standardized to the age distribution of the study population.

Abbreviations: CKD, chronic kidney disease; HIV, human immunodeficiency virus; NAFLD, nonalcoholic fatty liver disease; SD, standard deviation.

^a^Value is not age-adjusted.

**Figure 1.**
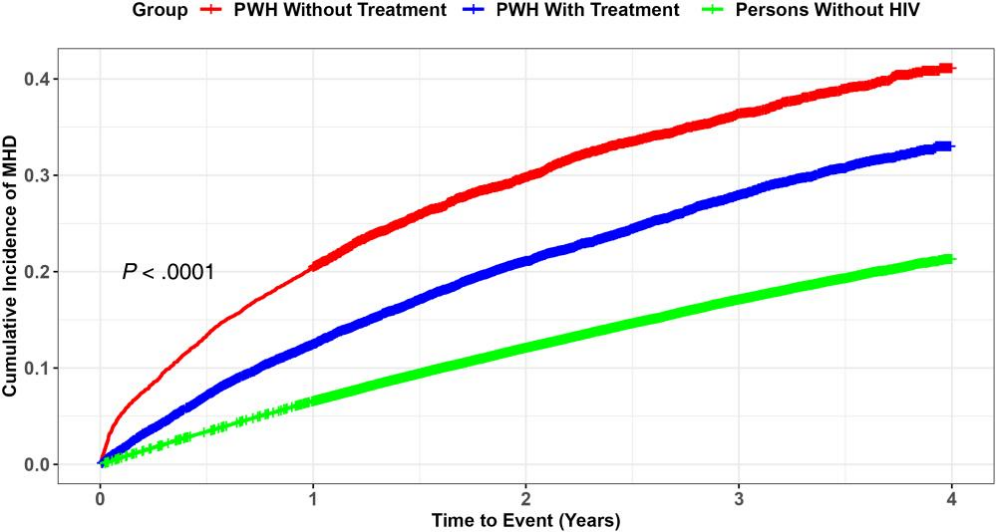
Cumulative incidence of mental health disorders (MHDs) among the human immunodeficiency virus (HIV) population. The incidence is higher in people with HIV (PWH) not on treatment, followed by PWH on treatment and lower in persons without HIV.

Among men, the unadjusted incidence rates of MHDs were higher among PWH without treatment (180.4 per 1000 PY) as compared to those without any diagnosis of HIV (59.0 per 1000 PY), though there was not a statistically significant difference in unadjusted rates among women. PWH without treatment in the age group of 18–34 years had the highest unadjusted incidence rate of MHDs (209.3 per 1000 PY) as compared to those without HIV (69.5 per 1000 PY) (Supplementary Table 1). PWH without treatment residing in the South region had higher incidence rates of MHDs (187.7 per 1000 PY) compared to those without HIV (65.0 per 1000 PY). We next examined multiple canonical risk factors for MHDs in these 3 cohorts (Supplementary Table 1). Among individuals with overweight/obesity, NAFLD, hypertension, diabetes, ischemic heart disease, CHF, and CKD, the unadjusted incidence rates of MHDs were consistently higher among PWH without treatment as compared to persons without HIV. The overall unadjusted incidence rate of MHDs was higher among PWH not on treatment (168.9 per 1000 PY) compared to those without HIV (63.5 per 1000 PY) (Table 2, Figure 1).

**Table 2.** Incidence Rates and Stratified Cox Proportional Hazard Models Hazard Ratios for the Association Between HIV and Mental Health Disorders in the MarketScan Database, 2016–2020

Variable	No HIV	HIV With Treatment	HIV Without Treatment
PY	618 605	36 782	16 493
Mental health disorder cases, No.	39 275	4174	2786
Incidence rate (95% CI) per 1000 PY^a^	63.5 (62.8–64.1)	113.5 (110.1–117.0)	168.9 (163.0–175.3)
Model 1	Reference	1.82 (1.76–1.88)	2.57 (2.47–2.67)
Model 2	Reference	1.85 (1.79–1.92)	2.70 (2.59–2.82)
Sensitivity analyses^b^			
Analysis based on propensity score strata	Reference	1.82 (1.77–1.88)	2.71 (2.61–2.82)
Excluding those taking antipsychotic, antidepressant, and antianxiety medications	Reference	1.82 (1.75–1.90)	2.71 (2.58–2.84)
Excluding individuals with common chronic conditions^c^	Reference	2.06 (1.97–2.15)	2.91 (2.76–3.06)

Model 1: Stratified by age (years) and sex (male/female).

Model 2: Model 1, plus further stratified by US region (Northeast, Midwest, South, West), place of residence (urban/rural), overweight/obesity, nonalcoholic fatty liver disease (NAFLD), hypertension, diabetes, dyslipidemia, stroke, ischemic heart disease, congestive heart failure, and chronic kidney disease (each yes vs no).

Abbreviations: CI, confidence interval; HIV, human immunodeficiency virus; PY, person-years.

^a^Unadjusted incidence rate per 1000 PY.

^b^Based on the full stratified model 2.

^c^Chronic conditions include obesity, NAFLD, hypertension, diabetes, dyslipidemia, stroke, ischemic heart disease, congestive heart failure, and chronic kidney disease.

After controlling for potential confounders in our fully stratified Cox model, PWH was associated with higher risk of developing MHDs (Table 2). Relative to those persons without HIV, the adjusted HR was 1.85 (95% CI, 1.79–1.92; *P* < .001) for PWH with treatment, and 2.70 (95% CI, 2.59–2.82; *P* < .001) for PWH without any treatment (Table 3). The analysis using the propensity score strata and excluding those taking antipsychotics, antidepressants, and antianxiety use yielded similar observed results (Table 2). Excluding participants with other common chronic conditions (overweight/obesity, NAFLD, hypertension, diabetes, dyslipidemia, stroke, ischemic heart disease, CHF, CKD) also generated similar results (Table 2). A similar pattern was observed when each individual category of MHDs was examined as an outcome (Table 3). Stronger associations between HIV and MHDs were observed in men relative to women, among those aged 18–34 years relative to those aged 55–63 years, and those with no overweight/obesity relative to obese individuals (*P*_interaction_ < .001 for all) (Supplementary Table 2).

**Table 3.** Stratified Cox Proportional Hazard Models Hazard Ratios for the Association Between HIV and Type of Mental Health Disorder in the MarketScan Database, 2016–2020 (n = 313 649)

Type of Mental Health Disorder	No HIV	Hazard Ratio (95% CI)	
		HIV With Treatment	HIV Without Treatment
Anxiety and related disorder	Reference	1.46 (1.38–1.54)	2.11 (1.97–2.25)
Major depression	Reference	2.62 (2.44–2.82)	3.50 (3.20–3.83)
Psychotic disorder	Reference	2.24 (1.38–3.62)	4.07 (2.48–6.70)
Other mood disorder	Reference	2.38 (2.00–2.84)	3.71 (3.03–4.56)
Other mental health conditions	Reference	1.94 (1.84–2.06)	3.04 (2.84–3.25)

*International Classification of Diseases, Tenth Revision, Clinical Modification* codes: Psychotic disorder (F20–F29), major depression (F32, F33), other mood disorder (F30, F31, F34–F39), anxiety and related disorder (F40–F48), and other mental health condition (F17–F19, F49–F69, F80–F89, F90–F99).

Based on the full stratified Cox proportional model: stratified by age (years) and sex (male/female), US region (Northeast, Midwest, South, West), place of residence (urban/rural), overweight/obesity, nonalcoholic fatty liver disease, hypertension, diabetes, dyslipidemia, stroke, ischemic heart disease, congestive heart failure, and chronic kidney disease (each yes vs no).

Abbreviations: CI, confidence interval; HR, hazard ratio.

## DISCUSSION

In this large-scale study of 313 539 individuals using the MarketScan claims database, we found that PWH have an increased risk for developing composite MHDs compared with persons without HIV. A similar pattern was observed when individual MHD diagnosis was examined as outcomes. These associations were independent of demographics and major chronic medical conditions. Treating HIV with ART was associated with lower risk of MHDs compared to those without treatment. To our knowledge, our study represents the first attempt to determine if treatment of HIV with ART is associated with lower risk of MHDs using large real-world data of the insured population in the US. HIV treatment was significantly associated with lowered MHDs but not to the level of subjects without HIV. The risk differed across sex, age groups, and overweight/obesity.

These findings reinforce the importance of integrating mental health screening and services in the treatment of PWH [25, 26]. Improved screening for MHDs among PWH, both in the context of HIV treatment and among those not on treatment, can provide opportunities for linkage to mental healthcare. Numerous models for HIV and mental healthcare integration exist, but fragmented care frequently leads to difficulties in implementation [27]. As the HIV epidemic in the US continues to age, MHD burden among PWH is likely to continue to grow [28] and yet MHDs in this population increase the risk of suicide deaths by nearly 100-fold [29].

Substantial evidence suggests that intersecting factors put individuals at risk of both HIV and MHDs [25]. Structural factors, such as poor housing and inadequate neighborhoods, are tied to both to HIV acquisition [30] and adverse mental health outcomes [31]. Additionally, it is plausible that there is a direct pathophysiological effect of HIV on the neurons since HIV is neurotropic, which may potentiate brain disorders including those of mental health [32]. Furthermore, MSM bear the highest burden of HIV in the US, with nearly 3 in 4 new HIV diagnoses among MSM [33], while simultaneously experiencing same-sex sexual stigma and discrimination that may lead to worsened mental health [34]. HIV-related stigma and the chronic stress of living with HIV may further exacerbate these vulnerabilities. HIV-associated stigma and loneliness partially explain depression among older HIV-positive adults, suggesting that interventions to reduce stigma and increase social support may help to combat increases in depression among PWH [35]. Work has shown that receipt of positive emotional and social support was inversely associated with mental health burdens among PWH [36]. Furthermore, among PWH, there are plausible biological mechanisms to suggest that chronic immune activation may lead to poorer mental health outcomes, particularly depression, among PWH [25]. Our findings suggest that this effect may be moderated by HIV treatment.

Previous work has shown that those with MHDs are less likely to engage in HIV care [37, 38] and have poorer adherence to treatment [39]. Our findings suggest that the reverse is true as well—those on HIV treatment are less likely to develop MHDs than those who are not on HIV treatment. These findings suggest that beyond integrating MHD treatment with HIV treatment, providing opportunities for linkage to HIV care among those seeking treatment for MHDs can help decrease HIV-related morbidity due to unsuppressed HIV [40].

### Study Strengths and Limitations

Strengths of our study include an analysis based on longitudinal data of a large sample of PWH and controls without HIV. To the best of our knowledge, it also is the first study to examine the association between HIV treatment and MHDs among a large sample of commercially insured patients using national real-world data. Notwithstanding, our study has several limitations that should be noted when interpreting the results. This is an observational study that used US medical claims data and therefore causality cannot be inferred. Our current study design included those who had continuous enrollment in their private insurance plan from 1 year before to 1 years after index date, thus creating some selection bias, but this continuous enrollment has enabled us to establish some boundaries for health history. Moreover, because of the complexity and lack of additional information from the database, we could not conduct analyses of specific ART regimens on the risk of developing MHDs for the present study. While all data were collected retrospectively, individuals were prospectively followed forward in time to determine the outcome of interest. We are unable to account for individuals with undiagnosed MHDs being potentially less likely to initiate ART; thus, our findings that PWH on ART were less likely to develop MHDs than those not on ART could be confounded by this unmeasured factor. We acknowledge that claims-based databases can misclassify patients based on misreporting or underreporting of diagnoses or medications. Moreover, the MarketScan database does not capture data on race or ethnicity, thus precluding any analysis to assess for confounding of the relationship between MHDs and HIV or HIV treatment status by race/ethnicity.

Despite these limitations, we believe this study provides new real-world evidence regarding the association between HIV treatment and MHDs using large real-world data of the US population.

## CONCLUSIONS

Findings from this large real-world study indicate that HIV is associated with increased MHDs risk, consistent with previous studies. Our study shows that among PWH, treatment with ART was associated with lower risk of MHDs compared to those without treatment. Our findings highlight the importance of ART among PWH to reduce their risk of adverse health outcomes, including MHDs, and improve quality of life.

## Supplementary Material

ofad555_Supplementary_Data

## Supplementary Data

Supplementary materials are available at *Open Forum Infectious Diseases* online. Consisting of data provided by the authors to benefit the reader, the posted materials are not copyedited and are the sole responsibility of the authors, so questions or comments should be addressed to the corresponding author.

## Notes

***Author contributions.*** D. M. B. and V. M. C. designed the research (project conception, development of overall research plan, and study oversight). D. M. B. performed data extraction, analyzed the data, and performed the statistical analyses. K. A. R. and D. M. B. wrote the first draft of the manuscript. All authors performed review and editing and have read and approved the final manuscript.
