# Supplementary material for: Human Immunodeficiency Virus (HIV) Treatment With Antiretroviral Therapy Mitigates the High Risk of Mental Health Disorders Associated With HIV Infection in the US Population
Source: Open Forum Infect Dis. 2023 Nov 7;10(11):ofad555. doi: 10.1093/ofid/ofad555 (PMC10686352; doi:10.1093/ofid/ofad555)

Supplemental Table 1| Subgroups analysis results showing the unadjusted incidence rates of mental health disorders, 95% CI per 1000 person-years of according to the three groups of HIV status.

Characteristics	No HIV	HIV with Treatment	HIV without Treatment
Sex			
Male	59.0 (58.3, 59.6)	116.3 (113.0, 120.2)	180.4 (173.0, 188.2)
Female	84.0 (82.2, 85.6)	100.9 (93.6, 108.8)	137.8 (127.3, 149.2)
Age groups, y			
18-34	69.5 (68.1, 70.9)	160.6 (150.0, 172.1)	209.3 (195.2, 224.3)
35-44	64.2 (62.8, 65.5)	123.7 (115.6, 132.4)	185.2 (171.3, 200.3)
45-54	60.3 (59.3, 61.3)	105.8 (100.6, 111.3)	154.1 (144.0, 164.9)
55-63	62.0 (60.6, 63.4)	95.5 (89.8, 101.5)	135.6 (124.4, 147.7)
Region of US, %			
Midwest	65.1 963.9, 66.4)	124.2 (113.4, 136.1)	182.8 (163.4, 204.5)
Northeast	59.5 (57.9, 61.1)	104.1 (96.8, 112.0)	125.8 (116.4, 136.9)
South	65.0 (64.1, 66.0)	113.2 (108.7, 117.9)	187.7 (178.6, 197.3)
West	57.2 (55.3, 59.1)	119.1 (110.2, 128.7)	198.0 (176.3, 222.2)
Place of residence			
Rural	69.0 (67.3, 70.8)	127.8 (118.7, 137.5)	190.6 (174.0, 208.8)
Urban	62.6 (61.9, 63.2)	110.9 (107.3, 114.7)	165.2 (158.6, 172.0)
Overweight/obesity			
No	62.2 (61.5, 62.8)	113.5 (110.0, 117.2)	168.8 (162.4, 175.5)
Yes	80.5 (77.9, 83.2)	112.7 (100.1, 127.0)	169.8 (149.3, 193.2)
NAFLD			
No	63.2 (62.6, 63.8)	113.7 (110.2, 117.2)	168.8 (162.6, 175.3)
Yes	90.4 (83.3, 98.1)	102.9 (81.2, 130.6)	175.2 (130.8, 234.6)
Hypertension			

No	61.2 (60.5, 61.9)	114.9 (111.0, 118.9)	168.2 (161.3, 175.4)
Yes	72.8 (71.3, 74.3)	108.5 (101.6, 115.9)	171.8 (158.2, 186.5)
Diabetes			
No	63.3 (62.6, 63.9)	115.9 (112.2, 119.6)	172.5 (165.9, 179.3)
Yes	65.6 (63.5, 67.8)	93.6 (84.5, 103.7)	137.3 (120.6, 156.3)
IHD			
No	63.3 (62.6, 63.9)	113.2 (109.7, 116.7)	168.4 (162.2, 174.9)
Yes	73.5 (69.1, 78.3)	124.1 (104.3, 147.7)	190.9 (152.0, 240.2)
CHF			
No	63.4 (62.7, 64.0)	113.0 (110.0, 116.5)	168.5 (162.3, 174.9)
Yes	82.9 (74.5, 92.2)	164.0 (126.5, 212.7)	209.0 (151.5, 288.6)
CKD			
No	63.4 (62.8, 64.1)	114.0 (110.6, 117.6)	169.3 (163.1, 175.8)
Yes	69.4 (62.5, 77.0)	98.9 (83.6, 117.1)	151.7 (117.0, 196.7)

Abbreviation: NAFLD: Non-alcoholic fatty liver; NAFLD: Non-alcoholic fatty liver; IHD: Ischemic Heart disease; CHF: Congestive heart failure; CKD: Chronic kidney disease.

Supplementary Table2| HRs of MHDs according to baseline HIV status, stratified by sex, age, and obesity

	No HIV	HIV with treatment	HIV without treatment	P-interaction*
**Sex**				<0.001
Men	1(ref)	2.05 (1.98, 2.13)	3.14 (3.00, 3.28)	
Women	1(ref)	1.23 (1.14, 1.34)	1.64 (1.50, 1.78)	
**Age groups (years)**				<0.001
18-34	1(ref)	2.30 (2.14, 2.47)	2.96 (2.75, 3.18)	
35-44	1(ref)	1.92 (1.78, 2.06)	2.85 (2.63, 3.10)	
45-54	1(ref)	1.79 (1.69, 1.89)	2.59 (2.41, 2.78)	
55-63	1(ref)	1.62 (1.51, 1.73)	2.24 (2.04, 2.45)	
**Overweight/obesity**				<0.001
No	1(ref)	1.90 (1.83, 1.96)	2.75 (2.64, 2.87)	
Yes	1(ref)	1.42 (1.25, 1.61)	2.00 (1.74, 2.30)	

Models based on the multivariable stratified Cox proportional hazard regression models.

*P-interaction was tested using the -2-log likelihood ratio (-2 LL)

Supplementary Table 3: Diagnosis codes of the International Classification of Diseases 10th editions, Clinical Modification (ICD-10-CM), used to describe baseline/preexisting clinical medical conditions.

Variables	ICD-10 codes
HIV diagnosis	B20, B21, B22, B23, B24
Mental health disorders	F10-F16, F17-F69, F80-F89, F90-F99
Overweight & obesity	E66
Non-alcoholic fatty liver disease	K74.60, K76.0, K76.89, K76.9, E88.89
Hypertension	I10-I13, I15
Diabetes	E08, E09, E10, E11, E13, E14
Ischemic heart disease	I20-I25
Congestive heart failure	I09.9, I11.0, I13.0, I13.2, I25.5, I42.0, I42.5–I42.9, I43.x, I50.x, P29.0
Stroke	I60, I63, I61, I62, I64-I67
Chronic kidney disease	N18
Dyslipidemia	E78

Supplemental Table 4: Antiretroviral therapy (ART) drugs used to identify HIV patients with treatment in the MarketScan database.

GENERIC DRUG NAME	DRUG CLASS
Abacavir	NRTI
Emtricitabine	NRTI
Lamivudine	NRTI
Tenofovir disoproxil fumarate	NRTI
Zidovudine	NRTI
Lamivudine/Zidovudine	NRTI
Abacavir/Lamivudine	NRTI
Zalcitabine	NRTI
Abacavir/Lamivudine/Zidovudine	NRTI
Emtricitabine/Tenofovir	NRTI
Didanosine	NRTI
Stavudine	NRTI
Efavirenz	NNRTI
Etravirine	NNRTI
Nevirapine	NNRTI
Rilpivirine	NNRTI
Delavirdine	NNRTI
Atazanavir	Protease Inhibitors
Darunavir	Protease Inhibitors
Fosamprenavir	Protease Inhibitors
Ritonavir	Protease Inhibitors
Saquinavir	Protease Inhibitors
Tipranavir	Protease Inhibitors
Amprenavir	Protease Inhibitors
Indinavir	Protease Inhibitors
Lopinavir/Ritonavir	Protease Inhibitors
Nelfinavir	Protease Inhibitors
Enfuvirtide	Fusion Inhibitors

Maraviroc	CCR5 Antagonists
Dolutegravir	Integrase Inhibitors
Raltegravir	Integrase Inhibitors
Elvitegravir	Integrase Inhibitors
Ibalizumab	Post-Attachment Inhibitors
Cobicistat	Pharmacokinetic Enhancers

Nucleoside Reverse Transcriptase Inhibitors (NRTIs)

Non-Nucleoside Reverse Transcriptase Inhibitors (NNRTIs)

Supplemental Figure 1| log-log survival curves for checking the PH assumption for three groups were almost completely parallel and PH assumption was satisfied.

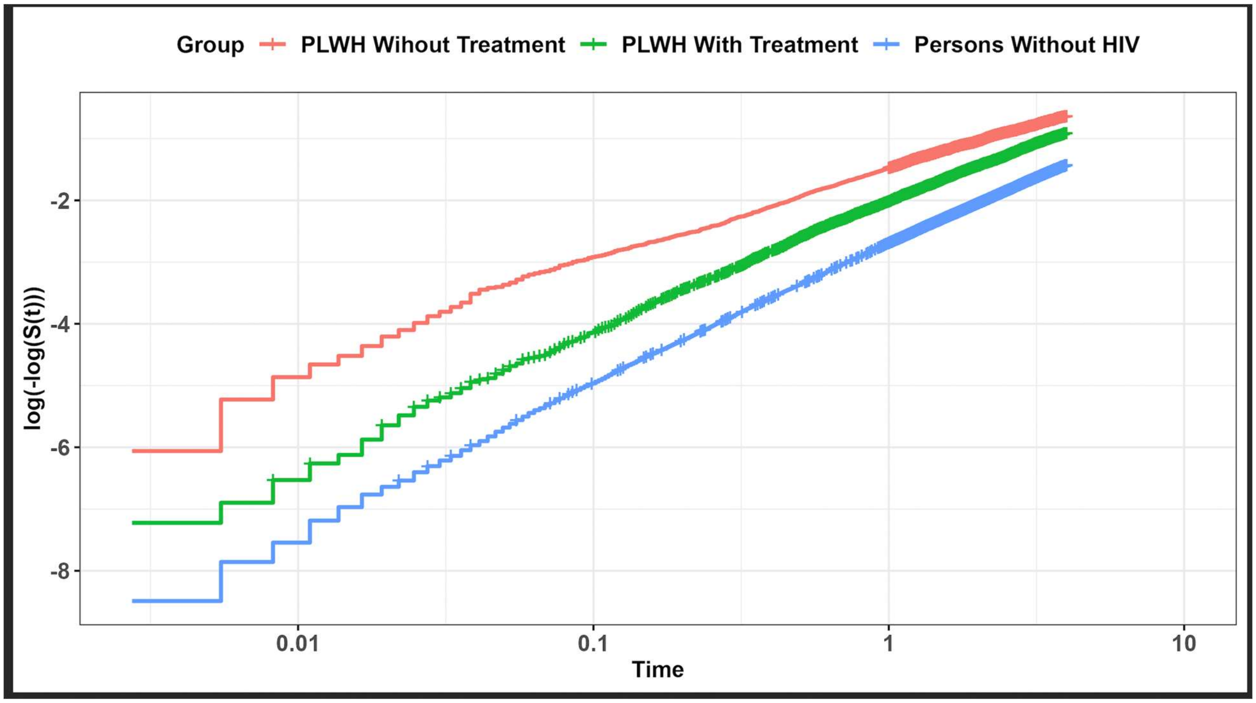

Supplement: ofad555_Supplementary_Data [file ofad555_supplementary_data.pdf]
